# Identification of biomarkers associated with diagnosis of postmenopausal osteoporosis patients based on bioinformatics and machine learning

**DOI:** 10.3389/fgene.2023.1198417

**Published:** 2023-07-03

**Authors:** Xinzhou Huang, Jinliang Ma, Yongkun Wei, Hui Chen, Wei Chu

**Affiliations:** ^1^Department of Orthopedics, 3201 Hospital of Xi’an Jiaotong University Health Science Center, Hanzhong, China; ^2^Department of Orthopedics, The First People’s Hospital of Jingzhou (First Affiliated Hospital of Yangtze University), Jingzhou, China; ^3^Department of Clinical Laboratory, The First People’s Hospital of Jingzhou (First Affiliated Hospital of Yangtze University), Jingzhou, China

**Keywords:** postmenopausal osteoporosis, machine learning, diagnosis, RNA regulatory pathways, Gene Expression Omnibus

## Abstract

**Background:** Accumulating evidence suggests that postmenopausal osteoporosis (PMOP) is a common chronic systemic metabolic bone disease, but its specific molecular pathogenesis remains unclear. This study aimed to identify novel genetic diagnostic markers for PMOP.

**Methods:** In this paper, we combined three GEO datasets to identify differentially expressed genes (DEGs) and performed functional enrichment analysis of PMOP-related differential genes. Key genes were analyzed using two machine learning algorithms, namely, LASSO and the Gaussian mixture model, and candidate biomarkers were found after taking the intersection. After further ceRNA network construction, methylation analysis, and immune infiltration analysis, ACACB and WWP1 were finally selected as diagnostic markers. Twenty-four clinical samples were collected, and the expression levels of biomarkers in PMOP were detected by qPCR.

**Results:** We identified 34 differential genes in PMOP. DEG enrichment was mainly related to amino acid synthesis, inflammatory response, and apoptosis. The ceRNA network construction found that XIST—hsa-miR-15a-5p/hsa-miR-15b-5p/hsa-miR-497-5p and hsa-miR-195-5p—WWP1/ACACB may be RNA regulatory pathways regulating PMOP disease progression. ACACB and WWP1 were identified as diagnostic genes for PMOP, and validated in datasets and clinical sample experiments. In addition, these two genes were also significantly associated with immune cells, such as T, B, and NK cells.

**Conclusion:** Overall, we identified two vital diagnostic genes responsible for PMOP. The results may help provide potential immunotherapeutic targets for PMOP.

## Introduction

Postmenopausal osteoporosis (PMOP) is a systemic metabolic phenomenon of low bone density and microstructural changes in postmenopausal women due to decreased estrogen levels (Marcus, 2002; Black and Rosen, 2016). The pathogenesis is mainly due to the imbalance of bone turnover balance caused by the decrease in estrogen secretion. Specifically, ovarian function and estrogen levels decreased in postmenopausal women, which resulted in decreased bone formation and increased bone resorption during bone remodeling, leading to decreased bone mass, decreased bone density, and increased fracture risk (Sotozono et al., 2022). In addition, factors such as poor diet, lack of exercise, smoking, and alcohol consumption may all play a role in the pathogenesis of postmenopausal osteoporosis. Osteoporosis (OP) affects more than 200 million postmenopausal women worldwide (Zhao et al., 2019), which is still increasing. OP affects 35 percent of postmenopausal women in the United States and causes approximately 1.5 million fractures yearly (Kamienski et al., 2011; de Bakker et al., 2018). When a hip fracture occurs, limb function and quality of life are significantly reduced, and medical costs and annual mortality also gradually increase (Zhang et al., 2020). Globally, postmenopausal women over 50 have a 50% risk of fracture due to OP, but most of these are preventable (Eastell et al., 2016). It has been estimated that osteoporotic fractures cause an excess of mortality of 9% for women and 24% for men at 1 y postfracture, and 24% and 26% 5 y postfracture (Mullin et al., 2020), respectively. Changes in molecular and cellular regulatory characteristics and biochemical processes of PMOP patients have attracted great interest in the discovery of neonatal markers (Lewiecki, 2011). Despite numerous studies on PMOP, the molecular mechanism of pathogenesis is still not fully understood. PMOP with delayed treatment is associated with a higher fracture risk (Chen et al., 2016). It is known that early recognition of PMOP is essential for early treatment and can even change the outcome of OP-induced fractures (Stepan et al., 2019). Currently, areal bone mineral density measurements obtained by dual-emission X-ray absorptiometry (DXA) are considered to be the most important predictor of the fracture risk; bone mineral density (BMD) only reflects bone mass. In addition, DXA is a multi-step process that requires demographic information, patient location, correct image analysis, and human identification. More than 90% of DXA tests or reports one or more errors, and approximately 80% of errors are related to image data analysis. DXA test errors may potentially impact patient management (Messina et al., 2015). Therefore, using big data to analyze disease-causing molecular characteristics of patients is an effective strategy to screen biomarkers for potential diagnosis and treatment.

PMOP is closely related to the immune system (Arron and Choi, 2000). In addition to the direct negative effects of estrogen deficiency in bone, the indirect effects of altered immune status in postmenopausal women may lead to persistent bone destruction as postmenopausal women often exhibit chronic low-grade inflammatory phenotypes, altered cytokine expression, and altered immune cell profiles. Previous studies have shown that lymphocytes in PMOP patients are significantly reduced, especially B lymphocytes, and apoptosis may be the primary mechanism of osteocyte regulation in PMOP patients (Breuil et al., 2010). At present, many bone protective agents are undergoing clinical trials for PMOP, such as denosumab, zoledronate, and teriparatide (Kendler et al., 2018; Reid et al., 2018; Tsai et al., 2019), but there are still bone loss, secondary fractures, and other events after the treatments. At the same time, a large number of studies (Reppe et al., 2017) have confirmed that epigenetics is involved in the occurrence and development of PMOP, participating in the differentiation of various bone cells by affecting the expression of multiple signaling pathways and related regulatory proteins, and epigenetics may be used as a diagnostic marker.

Due to the heterogeneity of tissues or samples in independent studies, most gene array results were either limited or inconsistent, particularly focused on a single cohort study or the algorithm needed to be updated, resulting in poor repeatability and consistency of the results. The goal of the treatment of PMOP is to prevent and reduce the risk of fracture and improve the quality of life of patients. There is an urgent need to find the methods of early diagnosis and treatment to improve the prevention and control measures of PMOP, and reduce the burden of medical costs associated with the occurrence and progression of PMOP. Multiple machine learning methods and expression spectrum analysis techniques were used to optimize these shortcomings. In this study, a comprehensive bioinformatics was used to explore biomarkers and potential therapeutic targets related to PMOP, and clinical specimens were collected for experimental verification.

## Materials and methods

### Data sources

The postmenopausal osteoporosis (PMOP) data were collected from the Gene Expression Omnibus (GEO) database (http://www.ncbi.nlm.gov/geo/). The array-based gene expression profiles of monocytes from 20 PMOP and 20 normal controls were included in the GSE56815 dataset. Another profile of monocytes from 26 PMOP and 16 normal controls was included in the GSE56814 dataset. The gene expression profiles of B cells in blood from 10 PMOP and 10 normal controls were included in the GSE7429 dataset. The raw data in these four datasets were removed of their background and normalized using the RMA algorithm affy package (Gautier et al., 2004).

### Clinical specimens

A total of 24 female participants aging from 48 to 59, including 12 PMOP patients and 12 control volunteers, were recruited from Jingzhou No. 1 People’s Hospital. The PMOP patients were diagnosed based on the fragility of the fractures in the postmenopausal women (Camacho et al., 2016), who were excluded if they had the following diseases: cancer, thyroid, rheumatoid arthritis, diabetes, oral bisphosphonate treatment, use of hormonal treatment, or other metabolic diseases.

### Analysis of differential gene expression

First, the gene expression profiles were constructed for each sample of PMOP and controls. Two gene expression profiles (GSE56815 and GSE56814) were merged into an independent dataset. Then, the inter-batch difference was removed with the removeBatchEffect function, and the differentially expressed genes (DEGs) between the PMOP and control groups in the datasets (GSE56815 and GSE56814) were identified using the limma package in R software (Ritchie et al., 2015). Similarly, differentially expressed genes in the GSE7249 dataset were calculated using the limma package in the R environment. A screening threshold of |log2FC| > 1 and *p* < 0.05 were set to obtain DEGs between PMOP and controls. Finally, DEGs in PMOP were analyzed to obtain the common genes.

### Enrichment analysis

The gene set variation analysis (GSVA) software package was used to identify the gene sets that were differentially expressed between the PMOP groups and controls in biological processes and signaling pathways (Hänzelmann et al., 2013). The two reference gene sets, namely, c5. bp.v7.1. symbols.gmt and c2. cp.kegg.v7.1. symbols.gmt (Liberzon et al., 2015), were obtained from the MsigDB v7.1 database. GSEA was used to define DEGs in the KEGG pathway using the hallmark gene sets (h.all.v7.2. symbols) (Halvorsen et al., 2019). GSEA was also used to identify signaling pathways that were positive or negative correlated hallmarks in the PMOP patients relative to the controls. The enrichment results of GSEA were displayed by the fgsea software package in R (Subramanian et al., 2005). The screening condition with statistical significance enrichment results was *p* < 0.05.

### LASSO regression

The diagnostic markers for PMOP were screened by the most minor absolute shrinkage and selection operator (LASSO) logistic regression. After quality control, two gene expression matrices (GSE56815 and GSE56814) were merged into an independent dataset. As a training set, 75% of the samples in the independent dataset were randomly selected. As λ increases, LASSO often shrinks the regression coefficient to zero. As a validation set, 25% of the samples in the independent dataset were chosen. The model effect of the obtained diagnostic markers was shown based on this independent dataset. The glmnet package in R was used for the LASSO algorithm (Friedman et al., 2010).

### Gaussian mixture and logistic regression models

The DEGs were analyzed using the online site of STRING (https://string-db.org), and the protein–protein interaction (PPI) network based on the connected nodes in the network was displayed. In order to further understand and predict the cellular function and biological behavior of the identified genes, we used STRING with a confidence score greater than 0.4 as the significance truncation criteria. DEGs with connected nodes in the network were used to build a Gaussian mixture model (GMM) using the mclust package in R (Browne et al., 2012). The hierarchical aggregation clustering method was used for classification based on the Gaussian finite mixture model. GMM was used to classify the mRNA clusters. The combined models used to predict PMOP were constructed using the logistic regression analysis. The receiver operating characteristic (ROC) curves were constructed to evaluate the predictive value of the models using the area under the curve (AUC). The optimal model was selected for further analysis. Finally, the common intersection of genes in LASSO and GMM was considered.

### Prediction of target miRNAs

miRNAs targeted in the aforementioned cross genes were predicted with mirDIP. The top 1% of miRNAs scores (Tokar et al., 2018) were processed by Cytoscape V 3.7.1., and miRNAs targeting more than two genes were selected.

### Construction of ceRNA networks

In order to study the interaction and target-binding relationship among different types of ceRNA in PMOP, the online tool StarBase (version 3.0) was used to predict upstream molecules’ lncRNAs interacting with the selected miRNAs (Li et al., 2014). miRNAs were set as the initial target, and their corresponding miRNAs and lncRNAs were connected. The predicted lncRNA, miRNA, and mRNA-targeted binding relationships were visualized using ceRNA-targeting relationship analysis results.

### Single-sample gene set enrichment analysis (ssGSEA)

Different marker gene sets of different immune cells types were identified by Bindea et al. (2013). The ssGSEA function in the GSVA package was used to predict gene signatures expressed by the immune cell populations (Hänzelmann et al., 2013). Correlations between levels of infiltration by different types of immune cells and genes were identified based on Pearson’s correlation, and *p* < 0.05 was regarded as statistically significant.

### Methylation analysis

The GSE99624 dataset included array-based gene methylation profiles of whole blood from 28 patients and 10 healthy controls. The ChAMP package in R was used to display the data results (Tian et al., 2017). The limma package in R was used to assess the methylation levels in PMOP and healthy controls.

### Quantitative reverse transcription polymerase chain reaction (qPCR)

Bone tissue samples were collected from participants using aseptic techniques. After the bone tissue sample was flattened, it was put into a mortar and ground to powder at low temperature with appropriate liquid nitrogen. The bone meal was collected in the centrifuge tube, 1 mL TRIzol was added, thoroughly mixed, and refrigerated at 4°C overnight. After centrifugation at 12,000 rmp at 4°C for 5 min, the supernatant was absorbed. Then, 0.2 mL chloroform was added, centrifuged again for 15 min, followed by addition of isopropyl alcohol of equal volume for 15 min, and retained precipitation. Add 1 mL 75% ethanol to the precipitate and centrifuge for 5 min to maintain the sediment. The aforementioned steps were repeated twice, dried until translucent, and DEPC water was added to dissolve the RNA precipitates. Total RNA from each sample was isolated from the bone tissue using the TRIzol^®^ reagent (Ambion Life Technologies, Carlsbad, CA, United States). The sample RNA was used as a template for reverse transcription into cDNA using the HiScript II Q RT SuperMix in the qPCR Kit (Vazyme, Nanjing, China). Quantitative real-time PCR (qPCR) was performed using SYBR Green^®^ Premix Ex Taq™ (Vazyme, Nanjing, China). The cycle length of the two PCR groups was 40 cycles to amplify DNA products, and the annealing temperature was 58 °C. GAPDH was used as internal reference, and the 2^−ΔΔCT^ method was used to analyze the qPCR results. The used specific primer sequences are as follows: the ACACB-forward primer (ATG​TTC​AGG​CAG​GCT​CTC​TT), reverse primer (ATT​TCC​ACC​AGG​AAG​TCG​GT); WWP1-forward primer (CCC​GGC​AGA​CAT​TGT​TTG​AA), reverse primer (CTC​TCG​CTA​GGC​CAC​CAT​AA); and GAPDH-forward primer (GGG​AAA​CTG​TGG​CGT​GAT), reverse primer (GAG​TGG​GTG​TCG​CTG​TTG​A).

### Statistical analysis

The gene expression difference between the two groups was statistically analyzed by the ggpubr package of R software. Gene expression data and all statistical analyses used R 4.0.0 (https://www.r-project.org/), GraphPad Prism 7.0 statistical software, and SPSS 22.0. When it conforms to the normal distribution, the two groups were compared by two independent sample *t*-tests. The Wilcoxon test was used for comparison between the two groups when it did not conform to the normal distribution. *p* < 0.05 was regarded as statistically significant.

## Results

### Data preprocessing and DEG screening

**FIGURE 1:**
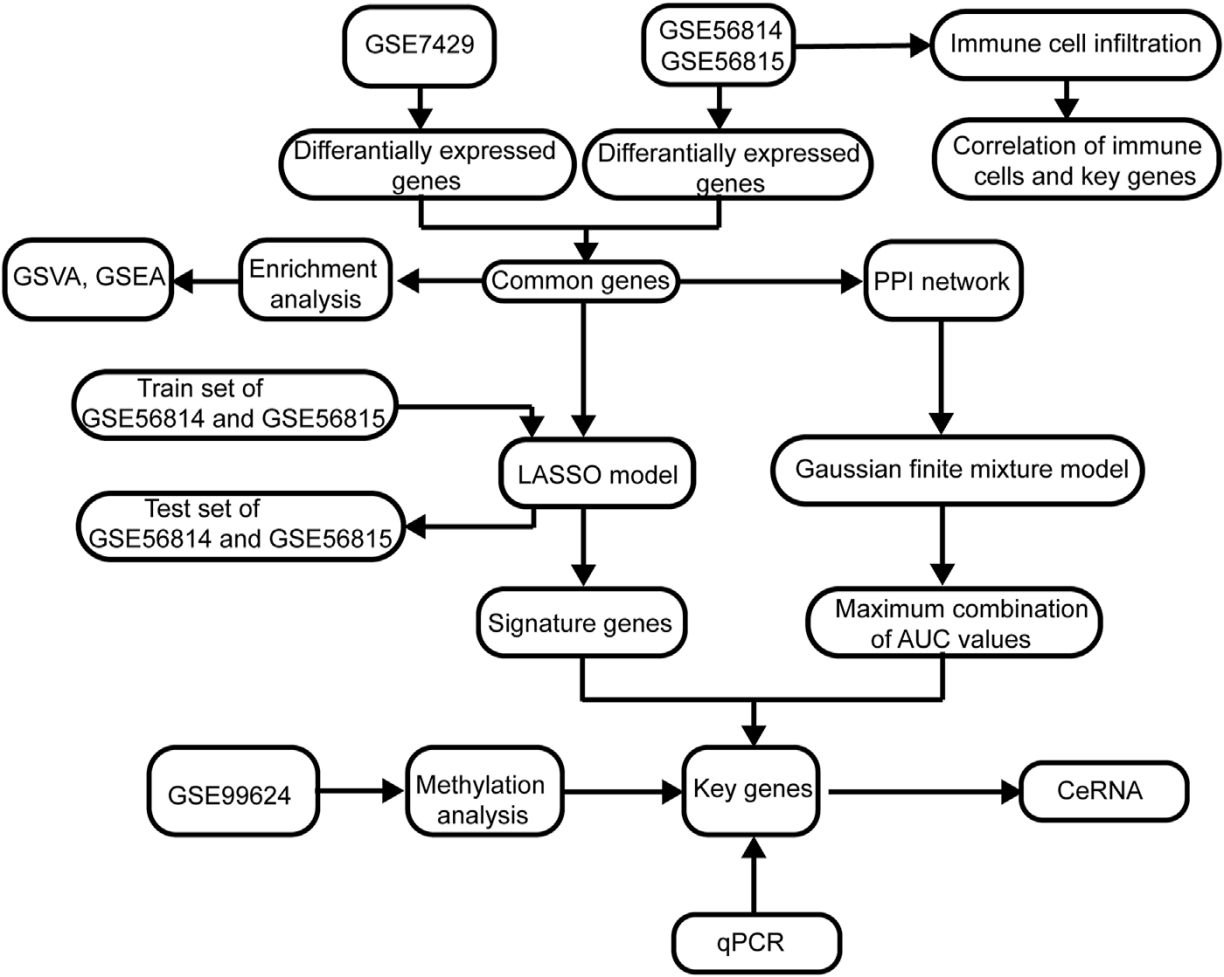
Flowchart of this study. The following datasets were used for identification of the potential diagnostic genes and mechanisms associated with the development of osteoporosis (PMOP): GSE56815, GSE56814, and GSE7429. Abbreviations: AUC, area under the receiver operating characteristic curve; GSVA, gene set variation analysis; PPI, protein–protein interaction; GSEA, gene set enrichment analysis; LASSO, least absolute shrinkage and selection operator.

**FIGURE 2:**
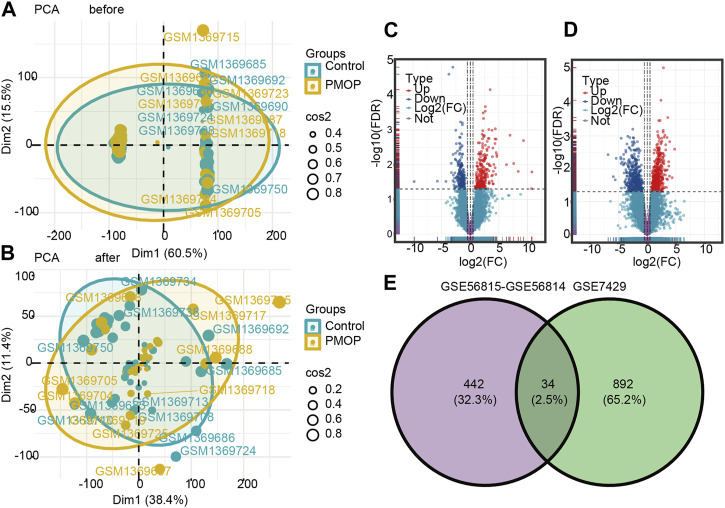
Two-dimensional PCA cluster plot before and after sample correction. **(A)** Two-dimensional PCA cluster plot of the GSE56815 and GSE56814 datasets before sample correction. **(B)** Two-dimensional PCA cluster plot of the GSE56815 and GSE56814 datasets after sample correction; yellow represents the osteoporosis (PMOP) group, and sky blue represents the normal control group. Volcano map and Venn diagram of differentially expressed genes (DEGs). **(C)** Genes differentially expressed between PMOP and controls in the GSE56815 and GSE56814 datasets. **(D)** Genes differentially expressed between PMOP and controls in the GSE7429 dataset. **(E)** Intersection of differentially expressed genes (DEGs) in the GSE56815, GSE56814, and GSE7429 datasets. The count on the left refers to DEGs unique to GSE56815 and GSE56814; the count in the middle, DEGs common to both datasets; and the count on the right, DEGs unique to GSE7429. DEGs were selected by |log_2_FC| > 1 and *p* < 0.05. Red represents upregulated differential genes, purple represents no significant difference genes, and blue represents downregulated differential genes. FC, fold change.

The flowchart of this study is shown in Figure 1. The removeBatchEffect method was used to remove the batch effect between the datasets. The merged datasets were normalized and presented in the form of the PCA cluster plot before and after normalization (Figures 2A,B).

### DEGs in PMOP

To identify PMOP associated genes, we analyzed genes differentially expressed between PMOP and control groups. A total of 476 DEGs in PMOP were obtained from the GSE56815 and GSE56814 datasets (Figure 2C; Supplementary Table S1) and 926 DEGs in PMOP from the GSE7429 dataset (Figure 2D; Supplementary Table S2). A total of 34 consistent DEGs were identified from the three gene expression profile datasets (Figure 2E; Supplementary Table S3).

### Enrichment of DEGs

**FIGURE 3:**
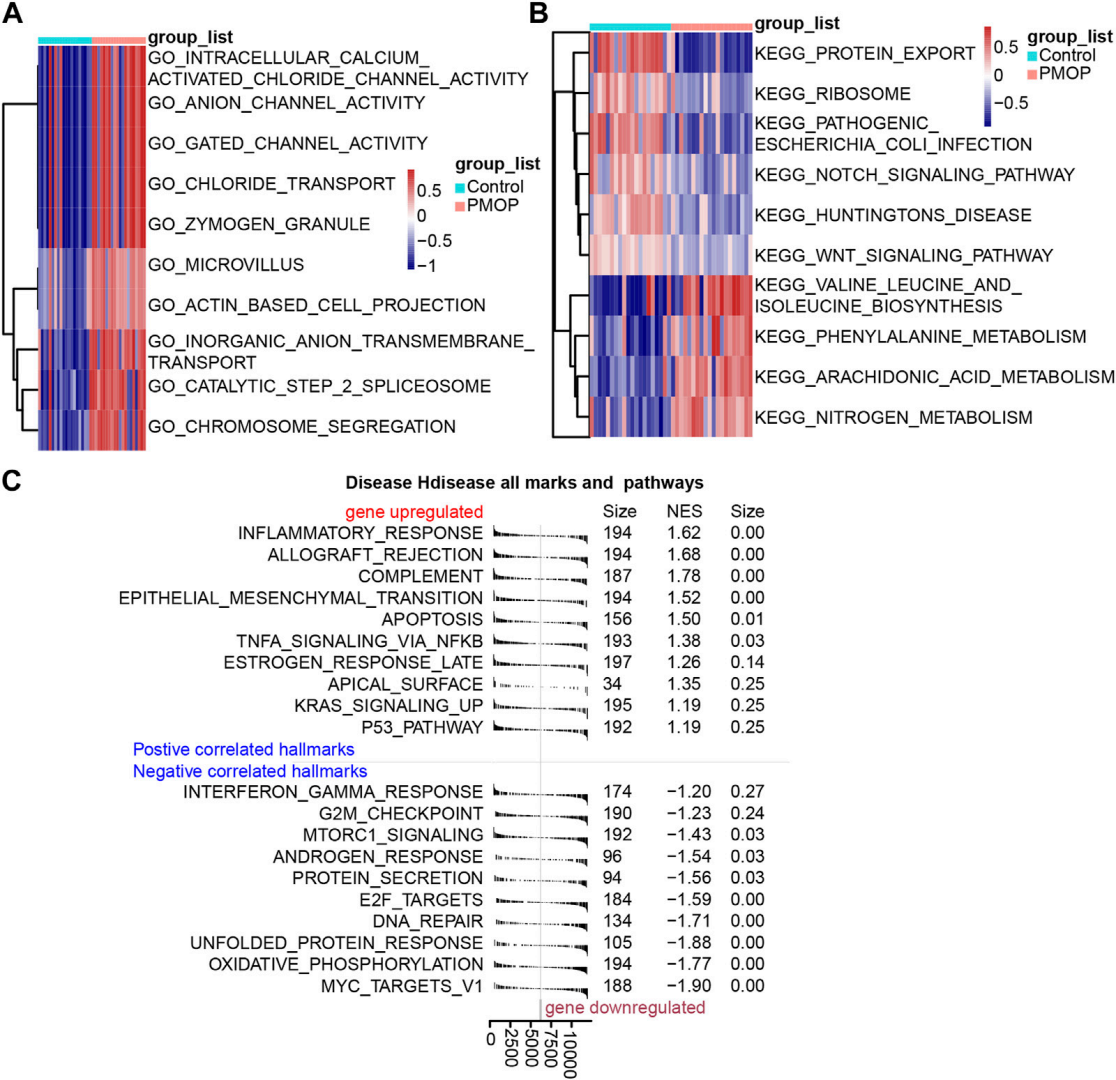
Biological functions and KEGG pathways enriched in DEGs. GSVA identified the biological processes **(A)** and KEGG pathways **(B)** between the DEGs of patients with PMOP. **(C)** DEGs involved in upregulated or downregulated KEGG pathways of GSEA results in PMOP patients relative to controls. *p* < 0.05 was considered statistically significant.

Based on the analysis of the GO enrichment, and KEGG pathway and GSVA of module genes, we found that intracellular calcium-activated chloride channel activity, actin-based cell projection, and anion channel activity of biological processes were enriched in the PMOP patients compared with the controls (Figure 3A). Among KEGG pathway results, valine, leucine, and isoleucine biosynthesis; arachidonic acid metabolism; and nitrogen metabolism were enriched in PMOP compared with controls. Conversely, protein export, Notch signaling pathway, and ribosomes were enriched in controls (Figure 3B). GSEA was used to study the potential biological processes and mechanisms of PMOP in different clusters (Figure 3C). The results revealed that the hallmarks of PMOP, including the inflammatory response (normalized enrichment score (NES) = 1.62), the estrogen response rate (NES = 1.26), KRAS signaling up (NES = 1.19), apoptosis (NES = 1.34), and TNFα signaling *via* NF-κB (NES = 1.37), were significantly and positively associated with PMOP. However, E2F targets (NES = −1.60) and MTORC1 signaling (NES = −1.43) were significantly and negatively associated with PMOP.

### Identification of genes with LASSO regression

**FIGURE 4:**
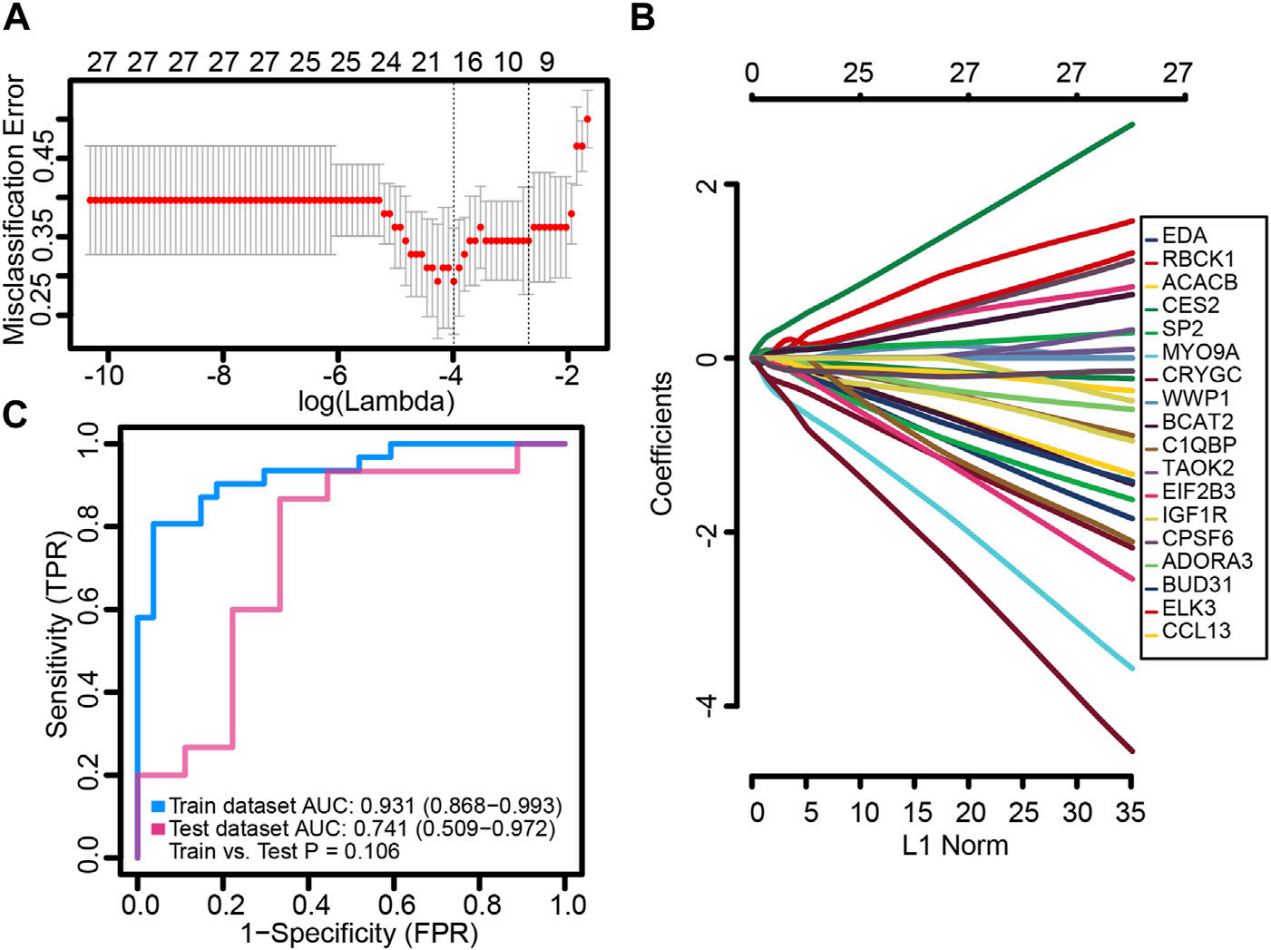
Potential key genes for the diagnosis of PMOP. **(A)** Gene signature selection of optimal parameter (lambda) in the LASSO model. **(B)** LASSO coefficient profiles of the 18 differentially expressed genes selected by the optimal lambda. **(C)** Receiver operating characteristic (ROC) curves of the gene signature in the training and testing sets of GSE56815 and GSE56814, respectively.

We randomly split the independent datasets (GSE56815 and GSE56814) into training (75%) and validation sets (25%). In the training set, common genes were used to construct the LASSO model. A λ value of 18 was selected as the best variable screening to determine the model gene that most accurately predicted PMOP (Figure 4A). Then, we drew a non-zero coefficient of 18 gene signatures (Figure 4B). The AUC values of gene signature in the training and validation sets were 0.931 and 0.741, respectively (Figure 4C).

### Identification of key genes associated with PMOP

**FIGURE 5:**
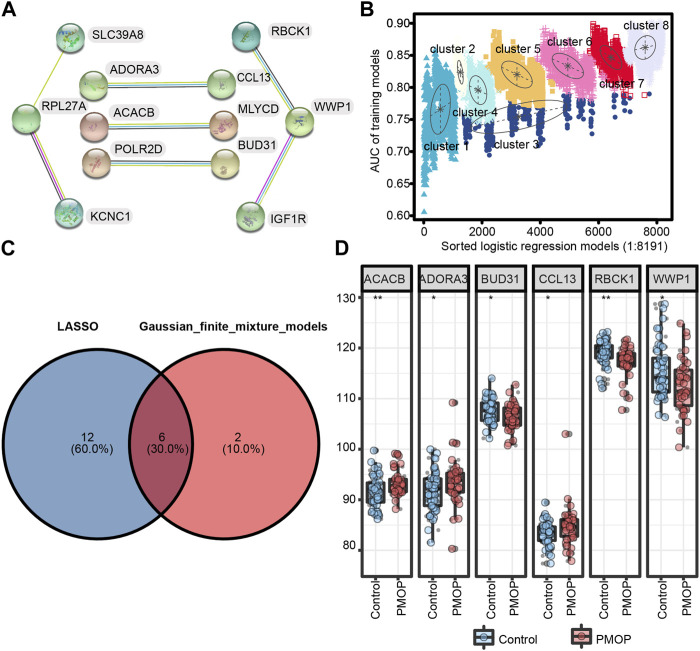
Screening and verification of diagnostic markers. **(A)** Protein–protein interaction (PPI) network of 12 genes; **(B)** pattern of the logistic regression model correlated with the AUC scores and was identified by a Gaussian mixture. There are eight clusters of 8,191 combinations. **(C)** Venn diagram shows the intersection of diagnostic markers obtained by the two algorithms. **(D)** Differential expression of key genes between PMOP patients and controls in GSE56815 and GSE56814, respectively. *p < 0.05; **p < 0.01.

First, we performed PPI network analysis of the DEGs with connection points (Figure 5A). The correlation between the expression values of 12 candidate mRNAs and the AUC values screened in PMOP analysis was evaluated by logistic regression analysis. The logistic regression model of 12 mRNA candidates produced 8,191 formulas. More importantly, decisive GMM-based clustering was used, with superior clustering performance (Li et al., 2008; Liu et al., 2016; Ficklin et al., 2017). Then, the gene sets were clustered using the GMM (instead of the 8191 formulas) and AUC algorithms into eight clusters. Afterward, among the eight clusters, the cluster with the highest AUC was selected as the signature for predicting the occurrence of PMOP. Hence, ACACB (acetyl-CoA carboxylase beta), ADORA3, BUD31, CCL13, RBCK1, WWP1 (WW domain-containing E3 ubiquitin protein ligase 1), SLC39A8, KCNC1, and RPL27A signatures showed an average accuracy of 0.85 using the GMM classifier in one of the 8,191 formulas (Figure 5B). The common genes ACACB, ADORA3, BUD31, CCL13, RBCK1, and WWP1 obtained by LASSO and GMM were selected (Figure 5C). Among them, ACACB, ADORA3, and CCL13 were upregulated in PMOP, while BUD31, RBCK1, and WWP1 were downregulated (Figure 5D). The expression values of these genes were validated based on samples from independent datasets (GSE56815 and GSE56814).

### Prediction of target miRNAs and construction of the co-expressed network

**FIGURE 6:**
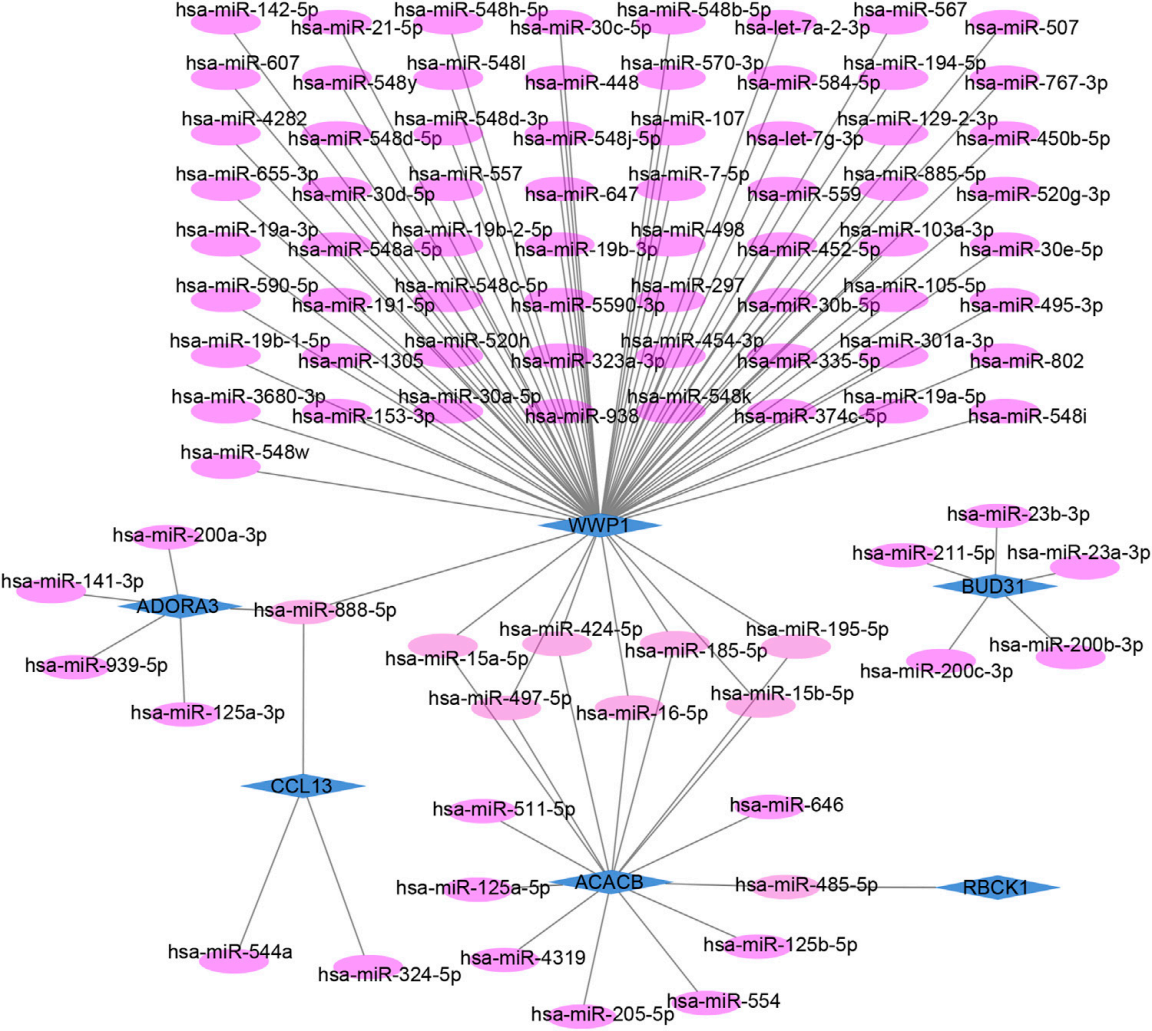
Co-expressed network of mRNAs and target miRNAs. The mRNA–miRNA co-expressed network was constructed by Cytoscape including 98 nodes and 102 edges. One node represents mRNA or miRNA, while one edge represents one interaction of mRNA and miRNA. Green diamonds represent the hub genes, and purple circles represent miRNAs.

The target miRNAs of hub genes were predicted using the mirDIP database. Finally, 101 target miRNAs of six specifically expressed hub genes were selected and 102 mRNA–miRNA pairs were determined. An mRNA and miRNA co-expression network consisting of 98 nodes and 102 edges was constructed by Cytoscape (Figure 6).

### Prediction of target lncRNAs and construction of ceRNA networks

**FIGURE 7:**
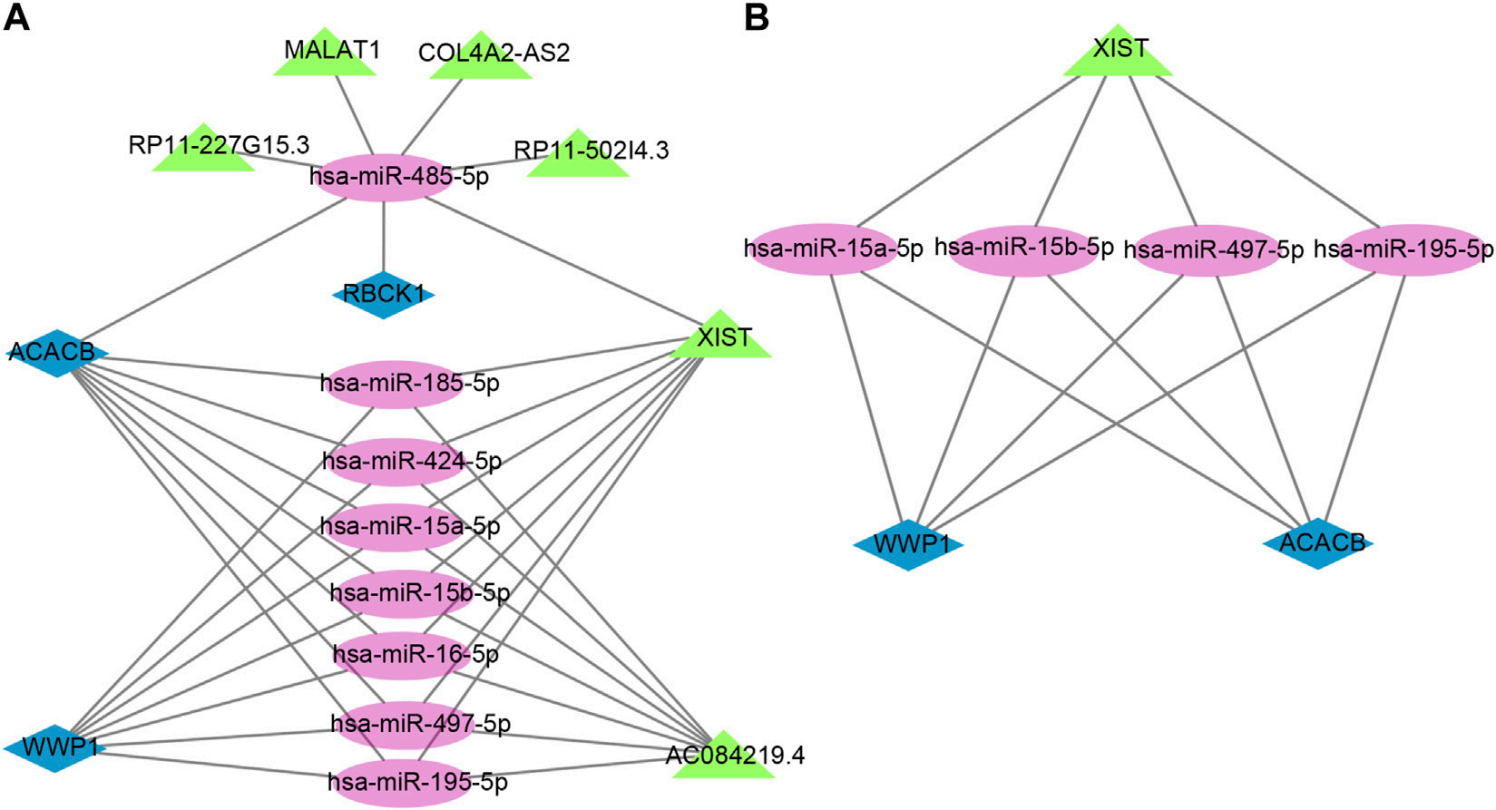
Potential RNA regulatory pathway for a ceRNA network. **(A)** ceRNA network of WWP1, RBCK1, and ACACB. **(B)** ceRNA network of WWP1 and ACACB. Blue diamonds represent the hub genes, purple circles represent miRNAs, and the green triangles represent lncRNAs.

The lncRNAs that interacted with selected miRNAs were predicted using starBase 3.0. The selection conditions were the human h19 genome and the highest reliability (very high stringency ≥5). Finally, we obtained five target lncRNAs of the target miRNAs of RBCK1, six target lncRNAs of the target miRNAs of ACACB, and two target lncRNAs of the target miRNAs of WWP1. Cytoscape software was used to construct and show ceRNA networks based on the prediction results (Figure 7A). Subsequently, we conducted literature retrieval and selected four reported miRNAs and two lncRNAs in bone metabolism for further analysis. We propose that XIST-miR-15a-5p/miR-15b-5p/miR-497-5p and miR-195-5p-ACACB/WWP1 might be potential RNA regulatory pathways to regulate the disease progression of PMOP (Figure 7B). Among them, ACACB and WWP1 were recognized as key genes and may be able to diagnose PMOP.

### Methylation marks in PMOP

To identify key genes that may be modified by methylation in patients with PMOP, we identified differentially methylated positions (DMPs) between PMOP and control groups in GSE99624 (Supplementary Figure S1A). DMPs were acquired and identified as methylation markers when the direction of delta beta values differed from that of normal DEG expression values. There were a total of 412,481 methylation markers that were identified (Supplementary Figure S1B). Among them the methylation levels of RBCK1 (cg25635139) were lower in PMOP than that in the control and that of ADORA3 (cg0054397) was higher in PMOP (Supplementary Figure S1C,D).

### Immune cell infiltration in PMOP

**FIGURE 8:**
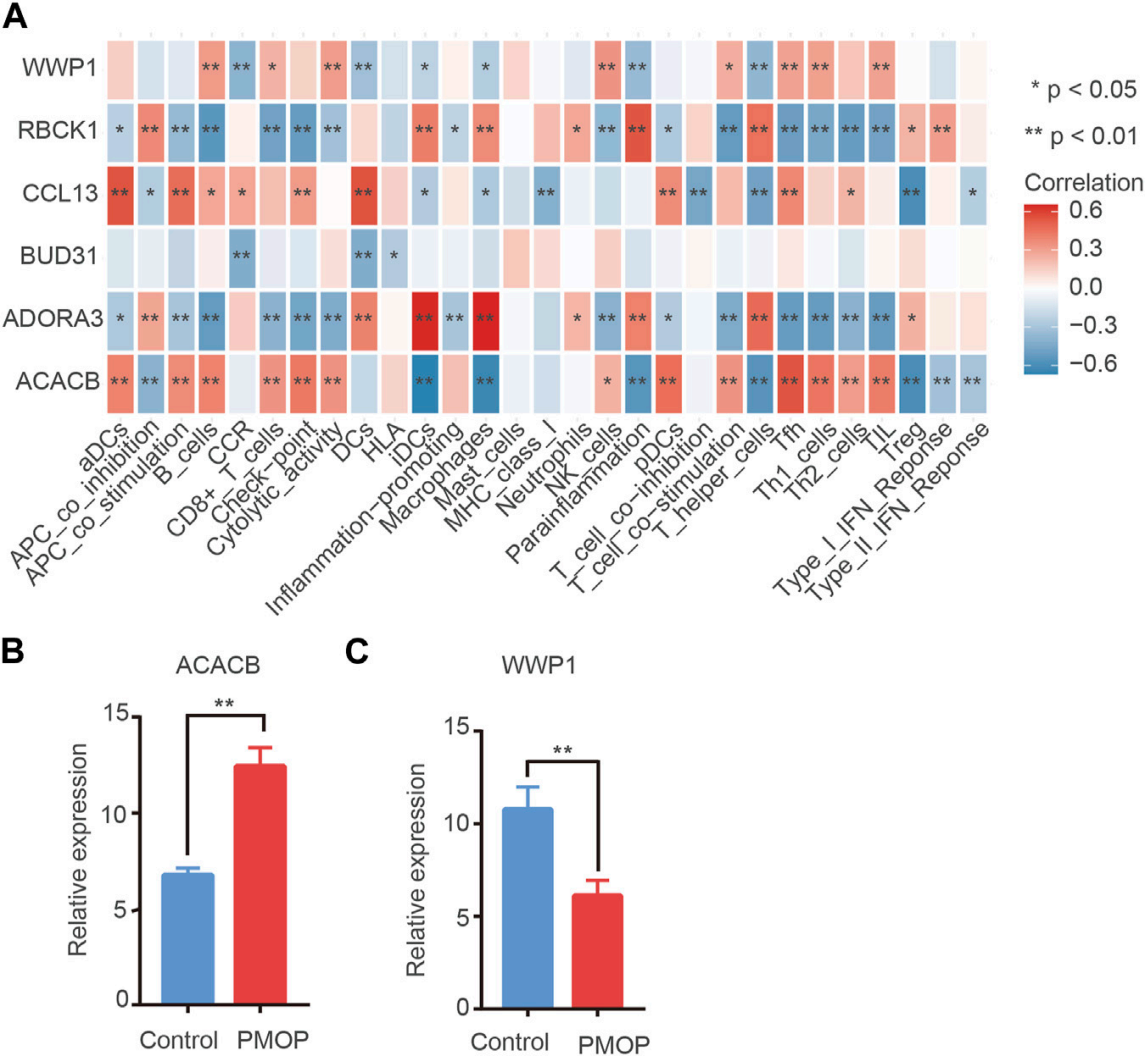
Immune cell situation in PMOP patients. **(A)** Correlation between key genes and immune infiltrating cells, based on Pearson correlation analysis. Red nodes represent positive correlations, and blue nodes, negative correlations. mRNA expression in PMOP patients. **(B–C)** QPCR results show that the expression levels of ACACB in PMOP patients were obviously higher than that of the healthy controls. However, the expression levels of WWP1 in PMOP patients were obviously lower than that of the healthy controls. Three independent experiments were performed. Similar results were obtained in each experiment, and the result of the representative experiment was presented. *p < 0.05; **p < 0.01. PMOP group versus the control group.

On one hand, the correlations between key genes and 24 types of immune cells were analyzed based on the ssGSEA function (Figure 8A). Correlation analysis showed that the level of ACACB was positively correlated with Tfh cells, the level of WWP1 was positively related to B cells and NK cells, and the level of ADORA39 was positively related to macrophages and T helper cells. In contrast, the level of RBCK1 was negatively related to Th1/Th2 cells. Therefore, hub genes might play an important role in the function of the immune cells. On the other hand, ssGSEA scores were calculated for these subtypes to generate immune cell interaction networks. The results showed that the differentially infiltrated immune cells were divided into four clusters (Supplementary Figure S2). In addition, significant cross-talks among the four clusters were found**.** They were further validated using qPCR analysis. Consistent with the prediction, the qPCR results showed that the expression levels of ACACB in bone tissue of the PMOP patients were higher than that of the healthy controls. The protein levels of WWP1 in the PMOP group were significantly decreased compared with that of the control group (Figures 8B,C).

## Discussion

The main feature of PMOP is that the rapid decline of the ovarian function and estrogen content in postmenopausal women leads to rapid bone loss and an increased risk of fracture, a systemic bone metabolic disease. Early recognition of PMOP can effectively prevent bone damage, which is the key to timely treatment and improving life quality. Clinicians have a great subjective initiative in diagnosing PMOP, and the operation process of DXA is tedious, especially in the aspects of patient positioning, correct image analysis, and manual recognition; there are significant errors, which becomes a problem for the accurate diagnosis of POMP. Clinically, bone markers, such as serum procollagen type I N-terminal propeptide (s-PINP), serum C-terminal telopeptide type I collagen (s-CTX), and urinary N-telopeptide (NTX), are commonly used to assess the bone level in bone diseases. However, S-CTX and S-P1NP have limitations due to the lack of normative reference population database and inadequate quality control standardization (Hackl et al., 2016). Moreover, these markers were influenced by multiple sources of variability, including daily intake and recent fractures (Eastell and Szulc, 2017). The diagnosis of PMOP is difficult due to the difference of the medical diagnosis level and the lack of effective biomarkers. In this context, studying new potential biomarkers for PMOP, especially early and effective diagnosis before the occurrence of a fracture, will prove important for both clinical practice and translational research.

In this study, we conducted extensive bioinformatic analysis of PMOP sequencing data to explore the molecular regulatory mechanism of PMOP. Importantly, a combination of the LASSO and Gaussian mixture models is used to identify potential diagnostic markers for PMOP. We further determined the potential biomarkers and methylation statuses associated with PMOP. We identified ADORA3, RBCK1, ACACB, WWP1, BUD31, and CCL13 as having a potential diagnostic role in PMOP, and the relationship between these potential diagnostic markers and immune cells. Moreover, ADORA3 and RBCK1 are modified by methylation, which may facilitate the diagnosis of PMOP.

Based on analysis of differential expression genes between PMOP and control samples, we identified 34 DEGs. GO enrichment analysis revealed that the intracellular calcium-activated chloride channel activity and actin-based cell projection were stronger in the PMOP samples than those in the control samples. KEGG pathway analysis of DEGs indicated that the amino acid metabolism, such as valine, leucine, and isoleucine biosynthesis, and the regulation of arachidonic acid metabolism, were stronger in the PMOP samples than those in the control samples. Conversely, protein export and ribosomes were enriched in controls. GSEA enrichment analysis also showed that DEGs were primarily enriched in the inflammatory response, immune response, and signal transduction. GO, KEGG, and GSEA enrichment analyses all showed that PMOP had strong inflammatory activation and signal transduction, which were the main causes of bone destruction caused by inflammation and estrogen imbalance. It is well known that bone destruction is the primary clinical manifestation of PMOP (Wehmeyer et al., 2016; Xu et al., 2017).

Based on the STRING website, we selected the differentially related genes for Gaussian mixture model analysis, nine genes were identified, and ROC analysis showed that they had a high diagnostic value. We combined the 18 genes analyzed by the LASSO model with the aforementioned genes, and the common genes selected are as follows: *ADORA3*, *RBCK1*, ACACB, *WWP1*, *BUD31*, and *CCL13*. Among them, *ADORA3* and *RBCK1* expression may be modified by methylation.

At present, *ADORA3* has not been reported in PMOP-related studies. However, in our research, *ADORA3* was upregulated in the PMOP. The gene *ADORA3* (also called A3AR) is a subtype of the adenosine receptor family (Guzman-Aranguez et al., 2014). Adenosine is called a retaliatory metabolite because it is expressed in response to injury or stress, when ATP is typically released outside the cell and metabolized to adenosine (Della Latta et al., 2013). The study found that ADORA3, an adenosine receptor regulated by pulsed electromagnetic field (PEMF), underwent CRISPR Cas9-mediated gene disruption. The mRNA and protein levels of osteocalcin and alkaline phosphatase in mouse preosteogenic cells were significantly decreased during the whole PEMF stimulation period (Kar et al., 2021). These results lead us to propose that the adenosine receptor ADORA3 plays an important role in PEMF-mediated osteoblast differentiation. A3AR is highly expressed in inflammatory cells and manifested in peripheral blood mononuclear cells in rheumatoid arthritis (Fishman and Cohen, 2016). Elevated extracellular adenosine levels are closely related to inflammatory conditions. Adenosine can regulate TLR-induced cytokine expression. A study of IL-12 expression in human myeloid APC found that both TLR and IL-12 expression were reduced in dendritic cells when adenosine receptor-mediated signal transduction was blocked. Both IFN-γ and IL-17, produced by T cells, were also significantly inhibited (van der Putten et al., 2019). In a rat model of diabetic nephropathy, ADORA3, as an upstream factor, when antagonized, can significantly inhibit the expression of downstream inflammatory cells and block the nuclear localization of nuclear factor kappa B (NF-κB) (Garrido et al., 2019). In chronic inflammatory diseases, PMOP is a common complication (Rao et al., 2018) and further bone destruction can be prevented by downregulating inflammatory cytokines. A3AR is highly expressed in inflammatory cells (Fishman and Cohen, 2016), so we believe that A3AR is a very effective biomarker for diagnosis, prevention, and treatment of PMOP.

RBCK1 interacts with two other ubiquitin ligases, namely, HOIP and SHARPIN, to form a linear ubiquitin chain assembly complex (LUBAC), which is a multi-immune signaling pathway (MacDuff et al., 2018). The NF-κB pathway is strictly regulated by various mechanisms including ubiquitination. Mutations in these regulatory pathways can lead to diseases (Steiner et al., 2018). As a key signaling pathway of inflammation and immune response, NF-κB signaling is attenuated and induces cell apoptosis and inflammation without RBCK1, HOIP, and SHARPIN (Tokunaga and Iwai, 2012). In breast cancer, the upregulated expression of RBCK1 mRNA is positively correlated with the expression of the estrogen receptor, and it is proposed that RBCK1 can be used as a diagnostic biomarker for breast cancer (Kharman-Biz et al., 2018). RBCK1 binds to ERα at the promoter of the estrogen-responsive gene *PS2* and regulates *PS2* levels (Donley et al., 2014). Our study found that *RBCK1* expression was downregulated in PMOP patients, and the decrease of estrogen in the patients after menopause may be one of the factors. It was further demonstrated that *RBCK1* positively correlated with the estrogen level. We believe that *RBCK1* is a new biomarker for the diagnosis of PMOP.

In addition, we constructed mRNA–miRNA co-expression networks and ceRNA networks of common genes to discuss the molecular pathogenesis of PMOP at the transcriptome level. Based on the ceRNA hypothesis, miRNAs in PMOP were screened out for further analysis. Between the target miRNAs of WWP1 and ACACB, the hsa-miR-497-5p expression was upregulated in stem cells from apical papilla. The hsa-miR-195-5p expression was downregulated in femoral head collapse (Li et al., 2017). Additionally, although miRNAs hsa-miR-15a-5p and hsa-miR-15b-5p have not been reported in PMOP, they have been reported to be upregulated in another bone disease, multiple myeloma (Li Z. et al., 2020).

Moreover, it has been reported that lncRNA XIST is upregulated in plasma and monocytes of patients with PMOP (Chen et al., 2019). Therefore, we propose that XIST - hsa-miR-15a-5p/hsa-miR-15b-5p/hsa-miR-497-5p and hsa-miR-195-5p - WWP1/ACACB might be potential RNA regulatory pathways to regulate the disease progression of PMOP.

WWP1 is an important regulatory factor related to human aging (Martin-Broto and Hindi, 2016). It has been shown that the exosome miR-19b can promote the differentiation of human bone marrow mesenchymal stem cells into osteoblasts by targeting WWP1 (Huang et al., 2021). It has been reported that WWP1 expression is decreased during osteogenic differentiation (Li Y. et al., 2020). As a negative regulator of the osteoblastic function, WWP1, when disrupted, may increase bone precipitation in osteoporosis patients (Tucker et al., 2018). It is suggested that WWP1 plays a vital role in the pathogenesis of osteoporosis. Consistent with this study, our study also found that WWP1 was downregulated in PMOP. ACACB, also known as ACC2, is an effective drug target for non-alcoholic steatohepatitis (Gao et al., 2020) and plays an essential role in regulating fatty acid oxidation (Wei et al., 2018). Some scholars point out that fatty acid metabolism is significantly related to changes in bone mineral density, and excessive intake of fatty acids will cause serious bone loss (Gong et al., 2021). In addition, the prevalence of osteoporosis in patients with the liver disease is increasing annually (Yang and Kim, 2021). Therefore, we believe that ACACB may play an essential role in the disease progression of PMOP and is considered a potential neonatal marker. As a splicing factor, BUD31 regulates gene expression through selective splicing (Hsu et al., 2015). This is consistent with several studies in which multiple splicing factors and events are associated with the development of osteoporosis (Fan and Tang, 2013; Shirakawa et al., 2016; Jia et al., 2019). CCL13 is a CC family chemokine also involved in many chronic inflammatory diseases (Mendez-Enriquez and García-Zepeda, 2013). The serum and synovial fluid levels of CCL13 in patients with knee osteoarthritis were higher than those in the control group. Moreover, the expression level was positively correlated with the imaging severity of OA, and the research team has proposed CCL13 as a biomarker for the progression of OA (Gao et al., 2015).

Bone immunology focuses on the relationship between the immune and skeletal systems. Postmenopausal women often exhibit chronic low-grade inflammatory phenotypes, altered immune cell profiles, and activated T cells that lead to elevated levels of pro-inflammatory cytokines, which may indirectly lead to bone destruction (Fischer and Haffner-Luntzer, 2021). For example, Th17 cells, a subtype of CD4 T cells, are bone-destroying cytokines (Tang et al., 2020). Studies have shown that estrogen regulates the abundance of dendritic cells (DCs) expressing IL-7 and IL-15 by inducing the apoptosis of Fas ligands and DCs (Cline-Smith et al., 2020). Estrogen induced antigen-independent production of IL-17A and TNF-α in memory T-cell subsets (Cline-Smith et al., 2020). Our immune infiltration analysis found that key genes were significantly correlated with some immune cells, such as DCs, macrophages, and T cells. In our cluster of immune cell types based on differential infiltration, the four clusters we identified showed obvious cross-talk relationships.

Our study is the first to study PMOP using a combination of LASSO and GMM. Further qPCR results showed that ACACB expression was upregulated in PMOP patients, whereas WWP1 expression was downregulated in the control group compared with the PMOP group. Our research also has some limitations. First, the sample size of the public database is small, which may lead to the deviation of the results. Second, it is necessary to further study whether the methylation of core genes is related to the occurrence and development of PMOP. In addition, we can study the correlation of key genes in immune cells, but we need to further study the content changes of immune infiltration between the two groups. Third, this study only verified the mRNA expression of key genes, and further study on the cell function of PMOP is needed.

## Conclusion

Multiple machine learning and experimental analysis revealed ACACB and WWP1 differential expression in postmenopausal osteoporosis bone tissues and infiltrated T, B, and NK cells. Our work identifies two genes that may be important for diagnosing PMOP. The mechanism of disease development of PMOP was further explored at the transcriptome level. With methylation analysis, *ADORA3* and *RBCK1* were identified as differentially methylated genes. All of them may serve as potential biomarkers to provide new insights into the diagnosis of PMOP. In addition, we propose that XIST - hsa-miR-15a-5p/hsa-miR-15b-5p/hsa-miR-497-5p and hsa-miR-195-5p–WWP1/ACACB were potential RNA regulatory pathways that control disease progression in PMOP.

## Data Availability

The datasets presented in this study can be found in online repositories. The names of the repository/repositories and accession number(s) can be found in the article/Supplementary Material.
