# Supplementary material for: Identification of biomarkers associated with diagnosis of postmenopausal osteoporosis patients based on bioinformatics and machine learning
Source: Front Genet. 2023 Jul 3;14:1198417. doi: 10.3389/fgene.2023.1198417 (PMC10352088; doi:10.3389/fgene.2023.1198417)
Supplement: Supplementary file 2 [file Table1.DOCX]

***Supplementary Material***

**Supplementary Figures**

**1. Supplementary Figure 1** **|** Screening of key methylation markers for PMOP in GSE99624. (**A)** Differentially methylated positions between PMOP and controls. Of all DMPs, 54.6% were hypermethylated and 45.4% were hypomethylated. Each feature color represents a different methylation site. (**B)** The methylation levels and the expression levels of methylation marks. Key genes are labeled. (**C, D)** Differences in key gene methylation levels between PMOP and controls. P for ADORA3 was 0.012; RBCK1, 0.004.

**2. Supplementary Figure 2** **|** Clustering and correlation of immune cell types based on infiltration levels. The size of each node represents the single-sample gene set enrichment analysis (ssGSEA) scores of each immune cell type, transformed by log10 (Log rank test P-value). Connections between immune cell types represent interactions between the two. The thickness of the line indicates the strength of the correlation, based on Pearson correlation analysis. Red lines represent positive correlations, blue lines, negative correlations. Immune cell cluster A is shown in yellow; cell cluster B, blue; cell cluster C, red; and cell cluster D, brown.**P* < 0.05, ***P* < 0.01.


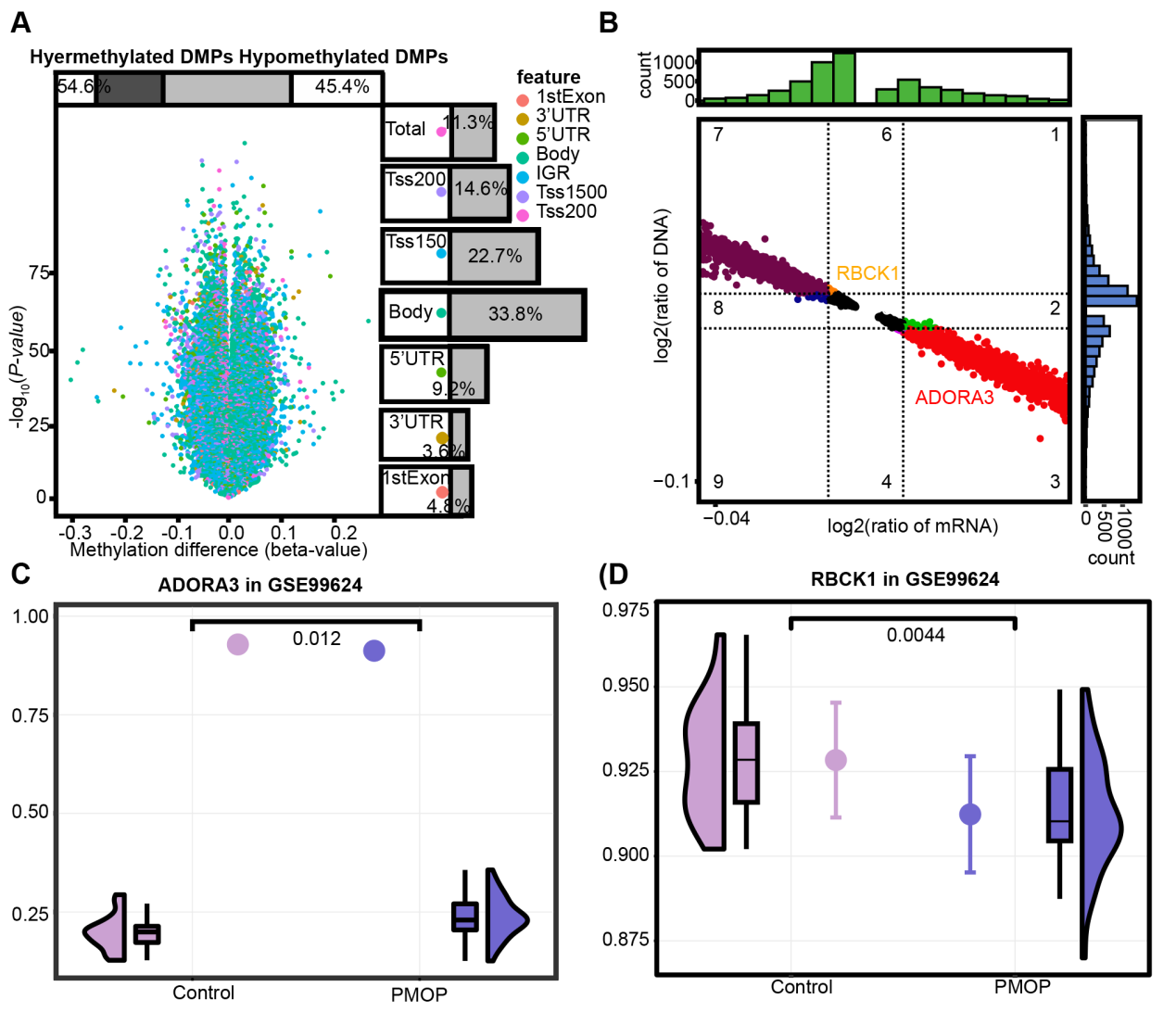


Supplementary Figure 1 | Screening of key methylation markers for PMOP in GSE99624.


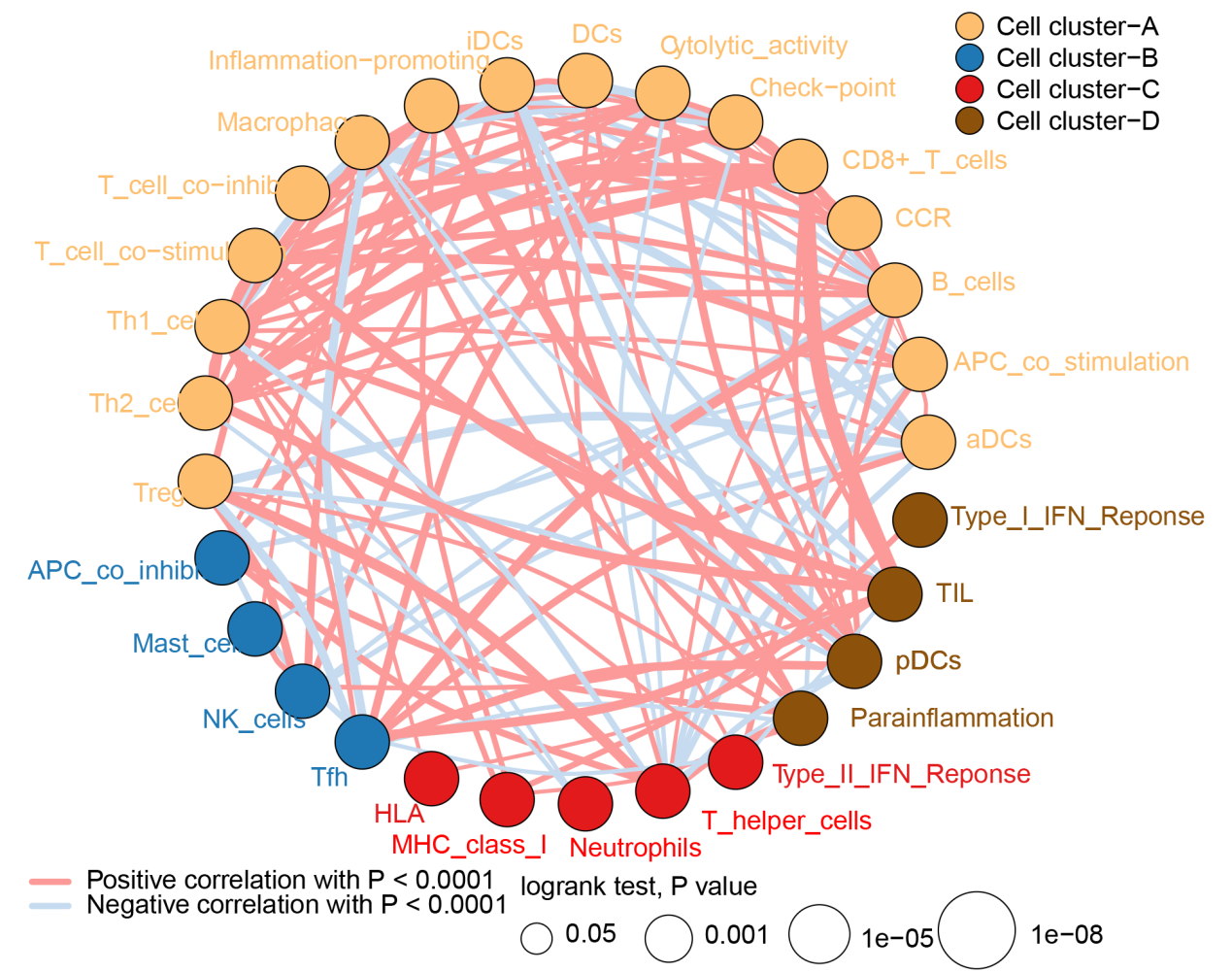


Supplementary Figure 2 | Clustering and correlation of immune cell types based on infiltration levels.
